# A History of Undergraduate Education for Public Health: From Behind the Scenes to Center Stage

**DOI:** 10.3389/fpubh.2015.00070

**Published:** 2015-04-27

**Authors:** Richard Kenneth Riegelman, Susan Albertine, Randy Wykoff

**Affiliations:** ^1^The George Washington University, Washington, DC, USA; ^2^Association of American Colleges & Universities, Washington, DC, USA; ^3^East Tennessee State University College of Public Health, Johnson City, TN, USA

**Keywords:** undergraduate public health education, history, specialized degrees, academic public health, public health pedagogy

## The Early Years – The Growth of Specialized Degrees

Education for Public Health traces its roots to the Welch–Rose report of 1915. The Welch–Rose report defined education for public health as applied graduate education primarily for professionals such as physicians, nurses, and engineers who needed academic education and the latest research to help them take on leadership roles in governmental public health (1).

The graduate and research focus of academic public health dominated the landscape for the better part of the twentieth century. Yet behind the scenes, changes were occurring that have led in the twenty-first century to new approaches to undergraduate public health education.

The Society for Public Health Education (SOPHE) was founded in 1950. As was the practice of the era, membership required a graduate degree. A decade later, SOPHE began admitting members with an undergraduate degree and practice experience (2). Undergraduate programs supported by SOPHE have included community health and school health.

The emergence of environmental health as a distinct field led to the development of environmental health programs at the bachelor’s degree level with a strong science emphasis.

In 1967, the National Environmental Health Science & Protection Accreditation Council (EHAC) was established. East Tennessee State’s undergraduate environmental health program became the first accredited undergraduate program (3).

When the Association of University Programs in Health Administration (AUPHA) was founded in the late 1940s, graduate degree programs formed the basis of eligibility for membership. Undergraduate programs were gradually added. By the late part of the century, AUPHA was offering a certification process for undergraduate health administration programs (4).

During the last half of the twentieth century, undergraduate programs in public health and related fields were developed that either did not qualify for membership in SOPHE, EHAC, or AUPHA, or chose not to pursue such membership. According to the Association of Schools and Programs of Public Health (ASPPH), by 1992, there were 45 institutions that were offering one or more undergraduate degrees in a public health related field. By 2000, this number had risen to 76 (5).

These early efforts to develop specialty degrees for undergraduates not only survived but have grown over the years. At the turn of the twenty-first century, they represented the major source of undergraduate education for public health.

## Undergraduate Education for Public Health – The Foundations of a New Movement

Efforts to frame education for public health as part of liberal arts education can be traced to the efforts of Abraham Lilienfeld, of Johns Hopkins. In the late 1970s, Dr. Lilienfeld wrote articles advocating development of “epidemiology 101” (6). This approach took root at Johns Hopkins where the School of Arts and Sciences in collaboration with the School of Public Health developed a major in public health. This major grew slowly in the early years but was well positioned for rapid growth and wide recognition in the twenty-first century (7).

In 1987, David Fraser, President of Swarthmore College and former CDC epidemiologist known for work on Legionnaire’s disease, wrote a ground breaking article in the New England Journal of Medicine titled: Epidemiology as a Liberal Art (8). For many who read the original article and others who read it decades later, the article served as an intellectual launching point for new ways to think about the role of epidemiology and public health as part of liberal arts or more broadly as liberal education^1^.

By the beginning of the twenty-first century, the student body for the MPH in most accredited Schools and Programs had changed dramatically. Recent bachelor’s degree graduates without any public health work experiences were enrolling in growing numbers and often made up the vast majority of MPH students. This fundamental demographic change set the stage for the full emergence of undergraduate public health education (9).

The early years of the twenty-first century saw a key turning point in the development of undergraduate education for public health. The 2003 Institute of Medicine report on the future of public health education included a key recommendation that “…all undergraduates should have access to education in public health” (10). This recommendation spawned key initiatives in 2003–2005 including the development of an Undergraduate Public Health Task Force by the then Association of Schools of Public Health (ASPH in 2013 became the ASPPH).

The growth of undergraduate public health education in accredited schools and programs proceeded rapidly in 2003–2005. By 2005, data collected by the Association of Schools of Public Health indicated that the majority of accredited Schools, which all offered graduate public health education, were also offering majors, minors, or individual public health classes for undergraduates. However, only a small fraction of all colleges and universities offered graduate education in public health, so there was still much more to do to bring education for public health to the majority of undergraduates.

## The Educated Citizen and Public Health Movement

As this growth occurred, it rapidly became apparent that the interest in public health at the undergraduate level was not limited to students who intended to pursue public health graduate degrees or a career in public health. Anecdotal evidence strongly suggested that students saw within the broader term “public health” a number of subjects of interest. Course work in global health was of particular interest as part of the then emerging focus on globalization and the importance of issues ranging from climate change to social justice.

A major turning point occurred in 2006 when the Council of Colleges of Arts and Sciences and the Association for Prevention Teaching and Research sponsored the Consensus Conference on Undergraduate Public Health (11). Key to the success of this effort was the participation of the Association of American Colleges and Universities (AAC&U) and the Association of Schools of Public Health. The Consensus Conference recommended a series of introductory “101” courses and the development of minors in public health for colleges and universities both with and without graduate programs in public health. Attendees also discussed the promise of incorporating public health courses and experiences into general education curricula that would reach virtually all undergraduates.

The participation of AAC&U coincided with the early years of AAC&U’s centennial defining initiative: Liberal Education and America’s Promise (LEAP). LEAP articulated a set of Essential Learning Outcomes delineating the best expectations for student learning in college. The ELOs, as they are known, describe the following four principal outcomes of an undergraduate education for the twenty-first century (12)^2^.

- Knowledge of human cultures and the physical and natural world – focused on engagement with big questions both contemporary and enduring;
- Intellectual and practical skills – practiced extensively across the curriculum, in the context of progressively more challenging problems, projects, and standards for performance;
- Personal and social responsibility – anchored through active involvement with diverse communities and real-world challenges;
- Integrative and applied learning demonstrated through the application of knowledge, skills, and responsibilities to new setting and complex problems.

Association of American Colleges and Universities leaders soon came to see undergraduate education for public health as an ideal exemplar to illustrate the potential of the ELOs to stimulate change in undergraduate education. The ELOs provided the foundation for the emergence of what came to be called the Educated Citizen and Public Health (ECPH) movement. The goals of this movement were described by Susan Albertine, Nancy Alfred Persily, and Richard Riegelman in a 2007 article titled “Back to the Pump Handle: Public Health and the Future of Undergraduate Education.” They wrote: “We need citizens who can help as individuals to change social behavior and who are aware of the need for systemic health care, good nutrition, decent housing, and sustainable urban centers. We need to rely on leaders who are able to consider benefits and harms to groups, minority as well as majority, and to engage in systems thinking, understanding how multiple factors interact. These are abilities essential to citizenship for the health of the world” (13).

Perhaps as a sign of the times, undergraduate institutions, even those without graduate public health education, rapidly adopted majors as well as minors and public health courses in general education. By 2008, when AAC&U conducted a survey of undergraduate programs, they found over 130 institutions offering undergraduate public health curricula.

The ECPH efforts led to a series of publications in national media and endorsements that have helped institutionalize the move toward undergraduate education in public health. In 2009, the American Public Health Association passed a resolution endorsing undergraduate public health education (14). *Healthy People 2020* included objectives designed to encourage the growth of undergraduate education in public health (15).

The rapid growth of majors and minors in public health was occurring in 2011 as schools and programs of public health prepared to examine the future of education for public health in preparation for the 100th anniversary of the Welch–Rose report in 2015. The ASPH Framing the Future Task Force was convened in 2011 and its first product was the ASPH Undergraduate Public Health Learning Outcomes built on the ELOs and designed for all undergraduates (16).

In 2012, the ASPH Framing the Future Task Force formed an expert panel chaired by Dean Randy Wykoff to which developed what has come to be called the Critical Component Elements (CCEs) designed for all undergraduate majors in public health and related fields (17). The CCEs have now been accepted by the Council on Education for Public Health as the basis for accreditation of undergraduate public health programs in institutions with and without graduate degree programs in public health. CCEs have also encouraged the development of a continuum of public health education extending from associate degree through to graduate public health education^3^.

## Community Colleges and Public Health – The Newest Frontier

Growth in education for public health has occurred primarily in bachelor’s degree programs. To bring community colleges into the continuum of public health education, the by then renamed ASPPH Framing the Future Task Force, in collaboration with the League for Innovation in the Community College (the League), produced the Community College and Public Health report in 2014 (18). Co-chaired by Richard Riegelman and Cynthia Wilson from the League, the report recommended two “prototype curriculum models” Public Health: Generalist & Specializations including general public health, health education, health administration, and environmental health designed for articulation with bachelor’s degree programs. Health Navigator applied associate degree and academic certificate programs were also recommended to prepare students for the workforce^4^.

Key to this initiative’s continuing success is ongoing collaboration with public health academic organizations including the SOPHE and the Association of Environmental Health Academic Programs (AEPHP) as well as public health practice organizations including the Association of State and Territorial Health Officials (ASTHO) and the National Association of County and City Health Officials (NACCHO). ASPPH and AAC&U continue to play key roles in encouraging the development of education for public health in community colleges.

## New Steps

A number of issues are key to the enduring impact of undergraduate education for public health. Mechanisms for articulation of associate degrees to bachelor’s degrees and bachelor’s degrees to the MPH have not been fully developed. The need for, and, as appropriate, the process for, “certification” of individuals who have received an undergraduate degree in a public health discipline, remains as challenging and important as it is for those receiving graduate degrees.

Finding a balance between global public health learning within general and liberal education as advocated by the ECPH movement and the emphasis on professionalizing public health degrees remains a worthy challenge. Finally, the relationship between number of jobs available and the number of individuals, receiving both graduate and undergraduate degrees in public health will need to be followed closely as will the impact of bachelor’s degree graduates on the public health workforce.

Undergraduate public health education is already having an impact on the MPH. The MPH report of the ASPPH Framing the Future Task Force (19) recommends coordination of the MPH core with the bachelor’s degree CCEs and recommends that the MPH include a coherent specialization. Both of these basic changes can be seen as a response to the development of undergraduate public health education.

Since 2011, ASPPH has sponsored the Undergraduate Education for Public Health Summit bringing together undergraduate faculty and administrators from institutions with and without graduate public health programs. The Summit has demonstrated the continuing need to expand the base of education for public health beyond schools and programs with graduate public health education.

These future issues may be seen as the challenges of success. Undergraduate education for public health has been a game changer influencing both undergraduate education, in general, and public health education, in particular. The emergence of undergraduate education for public health is already shaping the view of public health for a broad spectrum of educated citizens.

## Conflict of Interest Statement

The authors declare that the research was conducted in the absence of any commercial or financial relationships that could be construed as a potential conflict of interest.

## Supplementary Material

The Supplementary Material for this article can be found online at http://journal.frontiersin.org/article/10.3389/fpubh.2015.00070

A timeline of the history of undergraduate education for public health is included in the journal’s supplementary materials, which accompanies this article.

## Footnotes

^1^http://www.aacu.org/resources/liberal-education

^2^http://aacu.org/leap/essential-learning-outcomes

^3^http://www.aspph.org/educate/#educational-models

^4^http://www.league.org/ccph/
