# Supplementary material for: A History of Undergraduate Education for Public Health: From Behind the Scenes to Center Stage
Source: Front Public Health. 2015 Apr 27;3:70. doi: 10.3389/fpubh.2015.00070 (PMC4410484; doi:10.3389/fpubh.2015.00070)
Supplement: Supplementary file 1 [file Data_Sheet_1.PDF]

Timeline Undergraduate Education for Public Health

1915- Welch-Rose Report- Initiation of formalized public health education focused on schools of public health with emphasis on graduate education (1)

1950 Society for Public Health Education (SOPHE) established- Initial membership required graduate degree, 1961 membership requirement broadened to include those with undergraduate degree plus experience (2)

1967 - National Environmental Health Science & Protection Accreditation Council (EHAC) established East Tennessee State School of Public Health undergraduate environmental health program becomes the first accredited undergraduate program (3)

1978- Teaching of Epidemiology 101: The New Frontier published by Abraham Lilienfeld of Johns Hopkins. Johns Hopkins' Undergraduate public health program subsequently established in arts and sciences and later becomes one of the largest majors (4,5)

1987- Epidemiology as a Liberal Art- published by David Frazer in New England Journal of Medicine brought national attention to the link between public health and liberal education (6)

2003 Institute of Medicine recommended that "...all undergraduates should have access to education in public health" led to rapid increase in majors and minors at Schools and Programs of Public Health (7)

2006 Consensus Conference on Undergraduate Public Health sponsored by undergraduate and public health educational associations- recommended introductory course work in public health be offered to all undergraduates as well as public health minors. (8)

2007- present Educated Citizen and Public Health Initiative- Collaborative efforts to encourage undergraduate public health education by the Association of American Colleges and Universities (AAC&U), the Association of Schools and Programs of Public Health (ASPPH) (9,10)

2008-9 National publications provided wide visibility for educated citizen model of undergraduate public health education including theme issues of American Journal of Preventive Medicine, Association of American Colleges and Universities' publication Peer Review and front page article in the Washington Post (11,12,13)

2009 American Public Health Association (APHA) passed resolution endorsing undergraduate public health education at 2-year and 4-year colleges (14)

2010 Healthy People 2020 new objectives encouraged expanded undergraduate public health education at 4-year and 2-year colleges (15)

2011 ASPH Undergraduate Public Health Learning Outcomes- Expert panel made recommendations for implementing undergraduate public health education for all undergraduates through curricular and extracurricular education based on AAC&U's LEAP Essential Learning Outcomes (16)

2012 ASPH Critical Component Elements of an Undergraduate Major in Public Health- Expert panel made recommendations for all undergraduate public health and related majors (17)

2013 Council on Education for Public Health (CEPH) announced plan to voluntarily accredit undergraduate public health majors at institutions without graduate public health education (18)

2013-14 Community Colleges and Public Health project of the ASPPH Framing the Future Task Force and the League for Innovation in the Community College examines the roles of community colleges and makes recommendations for new programs as part of the continuum of public health education (19)

1. Welch WH, Rose W. Institute of Hygiene: a report to the General Education Board of Rockefeller Foundation. New York: The Rockefeller Foundation; 1915. Available at http://www.deltaomega.org/classics.cfm Accessed February 19, 2015.

2. Society for Public Health Education, SOPHE History available at http://www.sophe.org/SOPHE_History.cfm Accessed February 19, 2015.

3. National Environmental Health Science & Protection Accreditation Council (EHAC) http://www.ehacoffice.org/ Accessed June 5, 2013.

4. Lillienfeld AM, Garagliano F, Lillienfeld DE. Teaching Epidemiology 101: the new frontier. Int J Epidemiol 1978;7:377– 80.

5. Rienzi G. A major change. John Hopkins University Arts and Sciences Magazine, Fall/Winter 2006:23– 6.

6. Fraser DW. Epidemiology as a liberal art. N Engl J Med 1987; 316:309 –14.

7. Gebbie K, Rosenstock L, Hernandez LM. Who will keep the public healthy? Educating public health professionals for the 21st century. Washington DC: National Academy Press, 2003:144.

8. The Educated Citizen and Public Health: Report of the Consensus Conference on Undergraduate Public Health Education, Council of Colleges of Arts and Sciences, Williamsburg Va. 2007.

9. Association of American Colleges and Universities. The Educated Citizen and Public Health. http://www.aacu.org/public_health February 19, 2015.

10. Riegelman RK, Albertine S. Undergraduate public health at 4-year institutions: it's here to stay. Am J Prev Med 2011; 40(2):226-31.

11. American Journal of Preventive Medicine*,* Theme Issue on Undergraduate Public Health Education: 2008.

12. Association of American Colleges and Universities. Liberal education and undergraduate public health studies, theme issue. Peer Review Summer 2009.

13. Brown D. For a global generation, public health is a hot field. The Washington Post. 19 September, 2008; Sect A:1.

14. American Public Health Association- Integration of Core Public Health Education into Undergraduate Curricula Policy Statement 200915 Available at http://www.apha.org/policies-and-advocacy/public-health-policy-statements Accessed February 19, 2015

15. Healthy People 2020 Objectives for 4-year and 2-year colleges to increase public health education – Public Health Infrastructure Topic areas Available at http://healthypeople.gov/2020/topicsobjectives2020/objectiveslist.aspx?topicId=35 Accessed February 19, 2015.

16. ASPPH Undergraduate Public Health Learning Outcomes, Association of School and Programs of Public Health, Undergraduate Education for Public Health, Available at http://www.aspph.org/educate/#educational-models Accessed February 19, 2015.

17. ASPPH Critical Component Elements of an Undergraduate Major in Public Health, Association of Schools and Programs of Public Health, Available at http://www.aspph.org/educate/#educational-models Accessed February 19,2015.

18. History and Overview of Baccalaureate Accreditation. Council on Education for Public Health Available at http://ceph.org/constituents/programs-baccalaureate-level/history-and-overview/ Accessed February 19, 2015..

19. Community Colleges and Public Health Project, League for Innovation in the Community College, Available at http://www.league.org/ccph/ Accessed February 19, 2015
